# Brief communication: clinical jaundice is associated with poor adherence to boosted atazanavir among adults in an HIV clinic in Kampala, Uganda

**DOI:** 10.1186/s12981-026-00908-y

**Published:** 2026-07-04

**Authors:** Angella Nabakooza, Sharon Kitibwakye Nakamanya, Eva Agnes Laker, Elizabeth Katana, Noela Owarwo, Isaac Lwanga, Barbara Castelnuovo, Mohammed Lamorde, Freddy Kitutu Eric

**Affiliations:** 1 Department of Pharmacy, Makerere University School of Health Sciences, PO Box 7072, Kampala, Uganda; 2 Infectious Disease Institute (IDI), Makerere University College of Health Sciences, Kampala, Uganda; 3 School of Medicine, University of St Andrews, Fife St Andrews, Scotland, UK; 4 Sustainable Pharmaceutical Systems (SPS) Unit, Makerere University School of Health Sciences, Kampala, Uganda

**Keywords:** HIV, Boosted atazanavir, Clinical jaundice, Adherence, Antiretroviral therapy

## Abstract

**Background:**

Boosted atazanavir (ATV/r) is associated with clinical jaundice, which may affect ART adherence. However, the prevalence of clinical jaundice among people on ATV/r and its association with ART adherence is unknown in Uganda.

**Methods:**

We conducted a cross-sectional study among 236 adult patients on ATV/r at the Infectious Disease Institute. Clinical jaundice was assessed by self-report and observation; ART adherence was measured using the 8-item Morisky scale.

**Results:**

Period prevalence of clinical jaundice was 31.8%, while point prevalence was 21.1%. While 8.5% had poor adherence, clinical jaundice was a significant predictor of poor adherence (adjusted odds ratio = 3.03, 95% CI: 1.18–7.76, *p* = 0.021).

**Conclusion:**

Clinical jaundice is common among patients on ATV/r in Uganda and is associated with poor ART adherence. Strengthening pre-ATV/r (pre-switch) counselling and exploring safer regimen alternatives are required to improve outcomes.

## Introduction

According to Uganda’s consolidated guidelines for HIV/AIDS prevention and treatment[22], Dolutegravir (DTG) is the preferred anchor drug for adult first- and second-line ART. Boosted atazanavir (ATV/r) is the alternative second-line option, especially for patients who cannot use DTG or who fail non-nucleoside reverse transcriptase inhibitor or DTG-based first-line therapy. However, ATV/r is linked to indirect hyperbilirubinemia, which may progress to jaundice [2, 4].

Jaundice occurs when blood bilirubin levels exceed 3 mg/dL and may present clinically with yellowing of eyes, mucous membranes, and skin [17]. ATV/r-induced jaundice is due to inhibition of Uridine 5’-Diphospho-Glucuronosyl Transferase 1A1 (UGT1A1), an enzyme responsible for bilirubin conjugation [6]. Genetic polymorphisms such as UGT1A1*28 are common across African populations, with particularly high frequencies in equatorial African groups [8], increasing susceptibility to jaundice [18]. Clinical jaundice may lead to non-adherence or discontinuation of ART among people living with HIV (PLHIV) [6].

Previous studies have assessed ART adherence [12, 15, 23], and ATV/r-associated hyperbilirubinemia [4, 11, 16]. Clinical trials conducted in predominantly white populations report a point prevalence of clinical jaundice of 5–8% [11, 20], while a Hispanic cohort reported a period prevalence of 21% [4]. However, no studies from Africa, and none from Uganda, have described the prevalence of clinical jaundice, or its association with ART adherence among people taking ATV/r. This limits the applicability of findings from non-African populations, given the high frequency of UGT1A1 variants in equatorial African populations, which may increase susceptibility to ATV/r-related clinical jaundice. Understanding clinical jaundice in this context is relevant not only for HIV programs in Uganda but also for global treatment decisions where ATV/r remains a key second-line option. Recognizing and addressing visible ART side effects such as clinical jaundice is essential for sustaining adherence, improving counselling, and informing national pharmacovigilance and treatment guidelines. This study aimed to determine the prevalence of clinical jaundice and its association with ART adherence among adults on ATV/r attending an HIV clinic in Kampala, Uganda.

## Methods

### Study design and setting

A cross-sectional study nested within an existing HIV care cohort was conducted from 8th April to 5th May 2019 at the HIV clinic of Infectious Diseases Institute (IDI), Makerere University, located within Mulago National Referral Hospital, Kampala, Uganda. As of 2019 the clinic cared for over 8,300 PLHIV, including approximately 871 on ATV-based ART regimen, in line with Uganda MoH HIV guidelines and the WHO guidelines. Routine data were captured in both physical records and an electronic database, including information on ART regimens, adverse drug reactions, reasons for switching and stopping ART. A quality control officer routinely checked and validated the data entered.

As part of routine care, patients received counselling at ART initiation on adherence, potential adverse drug effects, and actions to take if symptoms occurred such as promptly returning to the clinic or contacting healthcare providers. Clinical assessments and management, including regimen review, were conducted during follow-up.

### Sample size

A sample size of 270 participants was calculated using Cochran’s formula [1], assuming 50% prevalence of clinical jaundice, 5% margin of error, and a 95% confidence interval (CI). This calculation was based on IDI clinical records for 2019, which indicated an estimated population of 871 PLHIV on ATV/r.

### Selection criteria and sampling

Participants were selected from the existing cohort of PLHIV receiving care at the IDI clinic and were eligible if they were HIV-positive adults (≥ 18 years) on ATV/r for at least 3 months. Those who were too ill were excluded. Consecutive sampling was used, and 242 participants were enrolled due to the time-bound recruitment period, representing 89.6% of the planned sample size.

### Data collection and data variables

Data were obtained from routinely collected data and primary data collected through a semi-structured, interviewer-administered questionnaire. The questionnaire was pre-tested among 10 adults on ATV/r at IDI clinic who were not part of the main study. The pre-test confirmed clarity and relevance of the questions, and no modifications to the tool were made. Collected data were entered into EpiData software and exported for analysis.

The main outcome was ART adherence, assessed using the 8-item Morisky Medication Adherence Scale (8-MMAS)[21], a widely used self-report tool for measuring medication adherence. Responses were coded and summed to generate a total score ranging from 0 to 8, with higher scores indicating better adherence. Adherence was categorized using standard 8-MMAS thresholds as high (8), medium (6–<8), and low (< 6). For the primary analysis, adherence was dichotomized into good (high and medium combined) and poor (low).

Independent variables included current or past clinical jaundice while on ATV/r, duration on ART, comorbidities (hypertension, diabetes and tuberculosis), social support, pill burden, disclosure, drug and substance abuse, stigma score and discrimination and socio-demographic characteristics (age, tribe, gender, religion, education level, employment, economic status, marital status). Clinical jaundice was assessed through self-report and visual examination of the sclera and buccal mucosa by trained study staff under natural light or bright indoor lighting. Distress due to clinical jaundice was graded using a visual analogue scale from 0 to10 [19]. Point 0 indicated that the person didn’t think about the clinical jaundice and 10 indicated that its presence maximally bothered them. Social support was assessed using the 3-item Oslo support scale, and numbers were assigned to each response. Total individual scores were categorized into 3: 3–8, 9–11, and 12–14, representing poor, moderate, and strong social support, respectively [10]. Stigma score and discrimination were measured using the 8-item Stigma Score for Chronic Illnesses (8-SSCI) [13]. Numbers were assigned to each response, and total individual scores were dichotomized; > 5 and < 5 indicated stigma and no stigma, respectively. Drug and substance abuse were assessed through self-report.

### Data analysis

Data were analyzed using Stata version 13. Descriptive statistics were used to summarize participant characteristics. The prevalence of clinical jaundice was calculated as point prevalence and period prevalence, expressed as percentages with 95% CI. Point prevalence was defined as the presence of clinical jaundice at the time of the study, while period prevalence was defined as having ever experienced clinical jaundice since initiation of the ATV/r regimen.

Bivariate logistic regression analysis was conducted to identify variables associated with adherence. Variables with p-values < 0.20 at the bivariate level (sex, alcohol use, clinical jaundice, stigma, and having discussed jaundice with a doctor) were considered for inclusion in the multivariable logistic regression model. Additional covariates of epidemiological relevance (age, duration on ATV/r, and social support), were also assessed. All variables were reported with crude odds ratios (cOR) and corresponding 95% CIs. A stepwise elimination approach was used to develop the final model, retaining variables with *p* < 0.05. Alcohol use was retained in the final model due to its clinical relevance.

Multicollinearity was assessed using variance inflation factors, and model fit was evaluated using the Hosmer–Lemeshow goodness-of-fit test. Effect modification by sex and age was assessed by including interaction terms. Predicted probabilities (marginal effects) of poor adherence by clinical jaundice status were estimated to aid interpretation of findings. Statistical significance was assessed at *p* < 0.05.

## Results

### Study participants

Of the 242 study participants enrolled, complete data from 236 respondents were analyzed (97.5% completion rate), and the mean age was 39.9 years (SD 10.8). Participants with clinical jaundice were younger (mean age 37.4 vs. 41.1 years, *p* = 0.0149) and more likely to be female (80.0% (40/50) vs. 60.3% (112/186), *p* = 0.003). No significant differences were observed in tribe, religion, education level, marital status, drug and substance abuse.

### Prevalence and characteristics of clinical jaundice

The point prevalence of clinical jaundice was 25.4% (60) (95% CI: 20.2–31.4), while period prevalence was 31.8% (75) (95% CI: 26.1–38.1) (Table 1). Clinical jaundice episodes varied in onset (*≥* 1week) and duration (*≥* 2weeks) after initiation of ATV/r and were often recurrent. Among those with clinical jaundice, 84.7% (47) reported that it minimally or did not bother them at all (visual analog score; 0–3) (Table 1). Only 2 participants (< 1%) discontinued treatment because of jaundice.

**Table 1:** Prevalence of clinical jaundice and ART adherence among 236 adults on ATV/r at IDI, Kampala, Uganda

Characteristic, *n* = 236	Frequency (*n*)	Percentage (%)	95% CI
Clinical jaundice (point prevalence)			
Yes	60	25.4	20.2 − 31.4 68.6–79.7
No	176	74.6	
Clinical jaundice (period prevalence)			
Yes	75	31.8	26.1–38.1
No	161	68.2	61.9–73.9
Visual analog score*, *n* = 70			
0–3	47	67.1	
4–7	16	16	
8–10	7	7.0	
Adherence (8-MMAS)			
Poor < 6	20	8.5	5.5–12.8
Good *≥* 6	216	91.5	87.2–94.5

*5 missing values

### ART adherence and associated factors

The majority (91.5%, *n* = 216) of the participants had good ART adherence. At bivariate analysis, sex, alcohol use, clinical jaundice, discussing jaundice with a doctor, and stigma and discrimination were associated with adherence (*p* < 0.20) (Table 2).

In multivariable logistic regression, clinical jaundice was independently associated with poor adherence (aOR = 3.03; 95% CI: 1.18–7.76; *p* = 0.021), while alcohol use showed a borderline association (aOR = 2.65; 95% CI: 0.93–7.65; *p* = 0.069). There was no evidence of multicollinearity, and the model demonstrated good fit (Hosmer–Lemeshow: χ² = 0.00, *p* = 0.9999). No statistically significant interaction was observed between clinical jaundice and sex or age (*p* > 0.05 for interaction terms).

The predicted probability of poor adherence was 5.5% (95% CI: 2.0%–9.0%) among participants without clinical jaundice, compared to 14.9% (95% CI: 6.9%–22.9%) among those with clinical jaundice, corresponding to an absolute increase of 9.3% points.

**Table 2:** Bivariate and Multivariable logistic analysis of the associations between variables and ART adherence among 236 adults on ATV/r at IDI, Kampala, Uganda

Variables, *n* = 236	Good adherence (*n* = 216), n (%)	Poor adherence (*n* = 20), n (%)	cOR (95% CI), *p* value	aOR (95% CI), *p* value
Sociodemographic characteristics				
Age in years			0.99 (0.99–1.03), 0.699	
Sex				
Male	75 (94.9)	4 (5.1)	1.00 (ref)	
Female	141 (89.8)	16 (10.2)	2.13 (0.69–6.59), 0.191*	
Tribe				
Muganda	124 (93.2)	9 (6.8)	1.00 (ref)	
Ankole	22 (91.7)	2 (8.3)	1.25 (0.25–6.19), 0.782	
Others	70 (88.6)	9 (11.4)	1.77 (0.67–4.67), 0.248	
Religion**				
Anglican	61 (91.0)	6 (9.0)	1.00 (ref)	
Catholic	69 (88.5)	9 (11.5)	1.33 (0.44–3.93), 0.611	
Muslim	37 (94.8)	2 (5.1)	0.55 (0.10–2.87), 0.477	
Pentecostal	45 (95.7)	2 (4.3)	0.45 (0.09–2.3), 0.344	
SDA	4 (100)	0	-	
Education level				
None	22 (95.6)	1 (4.4)	1.00 (ref)	
Primary	77 (89.5)	9 (10.5)	2.57 (0.31–21.4), 0.382	
Secondary	88 (92.6)	7 (7.4)	1.75 (0.20–14.9), 0.609	
Tertiary	29 (90.6)	3 (9.4)	2.28 (0.22–23.4), 0.449	
Marital status				
Married/Cohabiting	114 (91.2)	11 (8.8)	1.00 (ref)	
Separated/Widowed	63 (92.6)	5 (7.4)	0.82 (0.27–2.47), 0.728	
Single	39 (90.7)	4 (9.3)	1.06 (0.32–3.53), 0.921	
Alcohol				
No	184 (92.9)	14 (7.1)	1.00 (ref)	1.00 (ref)
Yes	32 (84.2)	6 (15.8)	2.5 (0.88–6.88), 0.085*	2.65 (0.93–7.65), 0.069
Clinical characteristics				
Clinical jaundice				
No	152 (94.4)	9 (5.6)	1.00 (ref)	1.00 (ref)
Yes	64 (85.3)	11 (14.7)	2.9 (1.15–7.34), 0.024*	3.03 (1.18–7.76), 0.021
Talked to the doctor about jaundice				
No	168 (93.3)	12 (6.7)	1.00 (ref)	
Yes	48 (85.7)	8 (14.3)	2.33 (0.90–6.03), 0.081*	
Perception				
Disclosure of HIV status				
No	13 (86.7)	2 (13.3)	1.00 (ref)	
Yes	203 (91.9)	18 (8.1)	0.58 (0.12–2.75), 0.49	
Stigma and discrimination (8 item score > 5)				
No	209 (92.1)	18 (7.9)	1.00 (ref)	
Yes	7 (77.8)	2 (22.2)	3.32 (0.64–17.16), 0.153*	
Pill burden (they take a lot of pills)				
No	143 (92.3)	12 (7.7)	1.00 (ref)	
Yes	73 (90.1)	8 (9.9)	1.31 (0.51–3.34), 0.577	

Variables with *p* < 0.20 at bivariate analysis were considered for inclusion in the multivariable logistic regression model. A stepwise elimination approach was used to obtain the final adjusted model, retaining variables independently associated with poor adherence at *p* < 0.05

*p value < 0.05

**1 missing value

## Discussion

This study found a high prevalence of clinical jaundice among PLHIV on ATV/r at IDI in Kampala, Uganda, with a significant association between clinical jaundice and poor ART adherence. These findings highlight how ART side effects may compromise adherence, leading to virologic failure, opportunistic infections, and increased mortality [3, 5].

The point prevalence of clinical jaundice (21.1%) was higher than in earlier trials, a randomized controlled trial (6–8%) and the CASTLE study (5%) [11, 20]. Similarly, the period prevalence of clinical jaundice (31.8%) exceeded that of a Hispanic cohort (21%) [4]. The difference in results may be attributed to the variation in UGT1A1 enzyme expression between the African, Hispanic and white populations. The UGT1A1*28 allele, which decreases the expression of the UGT1A1 enzyme, resulting in hyperbilirubinemia, is more prevalent in Africans (40%) than in Hispanics (38%) and whites (31%) [9, 18, 21].

Most participants reported good adherence, consistent with findings from a multicenter study conducted among adolescents in Uganda [15]. The good ART adherence may be attributed to good counselling skills, motivation schemes, and ART refill reminders by the IDI staff. However, adherence was likely to be impeded by the presence of clinical jaundice. Additionally, discontinuation of treatment due to clinical jaundice was rare, consistent with a prior study where less than 1% discontinued ART because of scleral icterus or jaundice [11]. This suggests that while clinical jaundice affects adherence, it rarely leads to treatment cessation since, its temporary. Clinical Jaundice episodes occurred more than one week after initiation of ATV/r, lasted more than two weeks, and were often recurrent, but most participants reported minimal concern. Although ART-related side effects are commonly reported as barriers to adherence, counseling and social support have been shown to influence patient perceptions and may influence how individuals respond to such effects [7].

### Implications for practice and policy

These findings suggest the need to strengthen counselling at ART initiation by preparing patients for the possibility of benign, reversible jaundice associated with ATV/r and clearly communicating that it does not necessitate treatment discontinuation. Routine assessment for jaundice during follow-up visits may help identify patients requiring additional adherence support. In addition, ART guidelines and patient education materials should incorporate practical guidance on the recognition and management of ATV/r-related jaundice, and healthcare providers should be supported to address patient concerns proactively.

### Future directions

Further research is needed to evaluate whether enhanced counselling and follow-up support can mitigate the impact of treatment-related side effects on adherence. Exploration of alternative regimens with a lower risk of jaundice, including unboosted atazanavir in appropriate settings, may also be warranted.

## Conclusion

Clinical jaundice is common among PLHIV on ATV/r and is associated with increased odds of poor ART adherence, posing challenges to achieving the 95-95-95 targets. Strengthening pre-ATV/r (pre-switch) counselling and exploring safer regimen alternatives are critical steps toward improving outcomes.

## Limitations of the study

Self-reported measures of jaundice and adherence may be subject to recall and reporting bias. Although validated tools were used, the 8-MMAS, Oslo social support scale, and 8-SSCI were not formally validated in this study population. Clinical jaundice was assessed through self-report and visual examination without laboratory confirmation using bilirubin levels, which may have resulted in misclassification, and inter-rater reliability was not assessed. The study did not incorporate objective adherence measures such as antiretroviral drug concentrations or viral load data, which may have provided additional insight into adherence patterns. In addition, some clinically relevant covariates, including HBV/HCV coinfection, hemolysis or anemia, genetic predisposition such as Gilbert syndrome, concomitant hepatotoxic medications, and nutritional status, were not available and may have resulted in residual confounding. The study was conducted at a single high-volume urban clinic, which may limit generalizability, although findings may be relevant to similar settings. Finally, recruitment was time-bound and the achieved sample size was slightly below the target, which may have reduced statistical precision.

## Data Availability

The datasets during and/or analyzed during the current study are available from the corresponding author on reasonable request.

## Abbreviations

8-MMAS: 8-item Morisky Medication Adherence Scale
8-SSCI: 8-item Stigma Scale for Chronic Illnesses
AIDS: Acquired immunodeficiency syndrome
AOR: Adjusted odds ratio
CI: Confidence interval
HIV: Human Immunodeficiency Virus
IDI: Infectious Disease Institute
OR: Odds ratio
PLHIV: People living with HIV
SD: Standard Deviation
WHO: World Health Organization

## References

[1] Bartlett II, Kotrlik JE, J.W. and, Higgins CC. Organizational research: Determing appropriate sample size in survey research. Inform Technol Learn Perform J. 2001;19(1):43.

[2] Bentu-Ferrer D, et al. Clinical pharmacology, efficacy and safety of atazanavir: A review. Expert Opin Drug Metabolism Toxicol. 2009;5(11):1455–68. 10.1517/17425250903321514. 19863454

[3] Biset Ayalew M. Mortality and its predictors among HIV infected patients taking antiretroviral treatment in Ethiopia: A systematic review. AIDS Res Treat. 2017;2017:1–10. 10.1155/2017/5415298. PMC5682904 29214077

[4] Bissio E, Lopardo GD. Incidence of hyperbilirubinemia and jaundice due to Atazanavir in a cohort of Hispanic patients. AIDS Res Hum Retroviruses. 2013;29(3):415–7. 10.1089/aid.2012.0233. PMC3581036 23121190

[5] Bukenya D, et al. What causes non-adherence among some individuals on long term antiretroviral therapy? Experiences of individuals with poor viral suppression in Uganda. AIDS Res Therapy. 2019;16(1):2. 10.1186/s12981-018-0214-y. PMC6340167 30665440

[6] Gammal RS, et al. Clinical Pharmacogenetics Implementation Consortium (CPIC) guideline for UGT1A1 and atazanavir prescribing. Clin Pharmacol Ther. 2016;99(4):363–9. 10.1002/cpt.269. PMC4785051 26417955

[7] Hlophe LD, et al. Barriers and facilitators to anti-retroviral therapy adherence among adolescents aged 10 to 19 years living with HIV in sub-Saharan Africa: A mixed-methods systematic review and meta-analysis. PLoS ONE. 2023;18(5):e0276411. 10.1371/journal.pone.0276411. PMC10194875 37200399

[8] Horsfall LJ, et al. Prevalence of clinically relevant UGT1A alleles and haplotypes in African populations. Ann Hum Genet. 2011;75(2):236–46. 10.1111/j.1469-1809.2010.00638.x. 21309756

[9] Kanestri V, et al. Clinical significance of the UGT1A1*28 allele detection in HIV-infected patients. J Int AIDS Soc. 2014;17(4 Suppl 3):19579. 10.7448/ias.17.4.19579. PMC4224789 25394086

[10] Kocalevent R-D, et al. Social support in the general population: standardization of the Oslo social support scale (OSSS-3). BMC Psychol. 2018;6(1):1–8. 10.1186/S40359-018-0249-9. PMC6050647 30016997

[11] McDonald C, et al. Clinical significance of hyperbilirubinemia among HIV-1-infected patients treated with atazanavir/ritonavir through 96 weeks in the CASTLE study. AIDS Patient Care STDs. 2012;26(5):259–64. 10.1089/apc.2011.0092. PMC3335103 22404426

[12] Mills EJ, et al. Adherence to HAART: A systematic review of developed and developing nation patient-reported barriers and facilitators. PLoS Med. 2006;3(11):2039–64. 10.1371/journal.pmed.0030438. PMC1637123 17121449

[13] Molina Y, et al. The Stigma Scale for Chronic Illnesses 8-Item Version (SSCI-8): Development, validation and use across neurological conditions. Int J Behav Med. 2013;20(3):450–60. 10.1007/s12529-012-9243-4. PMC3758464 22639392

[15] Nabukeera-Barungi N, et al. Adherence to antiretroviral therapy and retention in care for adolescents living with HIV from 10 districts in Uganda. BMC Infect Dis. 2015. 10.1186/s12879-015-1265-5. PMC4647509 26573923

[16] Panagopoulos P, et al. Hyperbilirubinemia in atazanavir treated HIV-infected patients: the impact of the UGT1A1*28 allele. Pharmacogenomics Personalized Med. 2017;10:205–8. 10.2147/PGPM.S107152. PMC5488765 28790862

[17] Ravindran R. Jaundice. Surg (United Kingdom). 2020;38(8):446–52. 10.1016/j.mpsur.2020.06.008.

[18] Ribaudo HJ, et al. Impact of UGT1A1 gilbert variant on discontinuation of ritonavir-boosted atazanavir in AIDS clinical trials group study A5202. J Infect Dis. 2013;207(3):420–5. 10.1093/infdis/jis690. PMC3537445 23148286

[19] Shillingford JP, Aitken CB. Measurement of feelings using visual analogue scales. Proc R Soc Med. 1969;62(10):989. 10.1177/003591576906201005. PMC1810824 4899510

[20] Squires K, et al. Comparison of once-daily atazanavir with efavirenz, each in combination with fixed-dose zidovudine and lamivudine, as initial therapy for patients infected with HIV. JAIDS J Acquir Immune Defic Syndr. 2004. 10.1097/00126334-200408150-00003. 15247553

[21] Turatti L, et al. Short communication: UGT1A1*28 variant allele is a predictor of severe hyperbilirubinemia in HIV-infected patients on HAART in Southern Brazil. AIDS Res Hum Retroviruses. 2012;28(9):815–8. 10.1089/aid.2011.0261. 22050734

[22] Uganda Ministry of Health. Consolidated guidelines for the prevention and treatment of HIV and AIDS in Uganda. Kampala: Ministry of Health, Uganda. 2022.

[23] Weiser S, et al. Barriers to antiretroviral adherence for patients living with HIV infection and AIDS in Botswana. JAIDS J Acquir Immune Defic Syndr. 2003;34(3):281–8. 10.1097/00126334-200311010-00004. 14600572

[24] Krousel-Wood M, Islam T, Webber LS, Re RN, Morisky DE, Muntner P. New medication adherence scale versus pharmacy fill rates in seniors with hypertension. *Am J Manag Care.* 2009 Jan;15(1):59-66. PMC2728593 19146365

